# Sphingosine 1-Phosphate Receptor 1 Modulates CNTF-Induced Axonal Growth and Neuroprotection in the Mouse Visual System

**DOI:** 10.1155/2017/6818970

**Published:** 2017-11-06

**Authors:** Sandrine Joly, Deniz Dalkara, Vincent Pernet

**Affiliations:** ^1^CUO-Recherche, Centre de recherche du CHU de Québec and Département d'ophtalmologie, Faculté de médecine, Université Laval, Québec, QC, Canada; ^2^INSERM, U968, Paris, France; ^3^Sorbonne Universités, UPMC Univ Paris 06, UMR_S 968, Institut de la Vision, Paris, France; ^4^CNRS, UMR_7210, 75012 Paris, France

## Abstract

The lack of axonal regeneration and neuronal cell death causes permanent neurological deficits in the injured CNS. Using the classical CNS injury model of optic nerve crush in mice, *ciliary neurotrophic factor* (CNTF) was found to stimulate retinal ganglion cell (RGC) survival and axonal growth, but in an incomplete fashion. The elucidation of molecular mechanisms impairing CNTF-induced axonal regeneration is paramount to promote visual recovery. In the present study, we sought to evaluate the contribution of *sphingosine 1-phosphate receptor 1* (S1PR1) to the neuroprotective and regenerative effects of CNTF. The transduction of retinal cells with adeno-associated viruses (AAV) allowed to activate CNTF/*signal transducer and activator of transcription 3* (Stat3) signaling and to modulate S1PR1 expression in RGCs. Our results showed that CNTF/Stat3 prevented injury-induced S1PR1 downregulation. Silencing S1PR1 in RGCs significantly enhanced CNTF-induced axonal growth in the injured optic nerve. In contrast, RGC survival was markedly decreased when S1PR1 was repressed with viral vectors. The level of phosphorylated Stat3 (P-Stat3), an intracellular mediator of CNTF, did not fluctuate after S1PR1 inhibition and CNTF stimulation. Collectively, these results suggest that S1PR1 acts as a major regulator of retinal neuron survival and restricts the RGC growth response induced by CNTF.

## 1. Introduction

Optic nerve axon damage is responsible for visual deficits in ophthalmic diseases such as glaucoma. In this ocular pathology, permanent vision loss is largely due to the lack of axonal regeneration in the adult optic nerve and retinal ganglion cell (RGC) death [1–3]. To study the mechanisms underlying RGC degeneration, the mouse optic nerve crush (ONC) is a model of choice [4]. After ONC, injured axons form stumps at the injury site and only a few of them are able to sprout shortly beyond [4]. The failure of axon regeneration is attributable to inhibitory molecules present in the environment of the injured optic nerve and to the intrinsically weak ability of adult neurons to activate a growth program [5–8]. However, it was demonstrated that experimental inflammation could place RGCs in a growth state and promote axonal growth in the crushed optic nerve [9, 10]. It was further shown that the injection of inflammatory compounds such as zymosan, a yeast cell wall extract, recruited macrophages and caused glial cell reaction in the retina [11]. Growth-promoting molecules released by immune and glial cells were identified in the retina. The secretion of oncomodulin by macrophages and neutrophils was proposed to mediate RGC growth during inflammation [12, 13]. In addition, *ciliary neurotrophic factor* (CNTF) is a potent inducer of RGC survival and axonal regeneration [14–17] that can be released by astrocytes and by specialized Müller glia [18]. Other cytokines of the CNTF family, such as *leukemia inhibitory factor* (LIF) and *interleukin 6* (IL-6), are also able to trigger RGC growth [19, 20]. The regenerative properties of CNTF are highly dependent on the intracellular activation of the *signal transducer and activator of transcription 3* (Stat3) transcription factor in RGCs [7, 21, 22]. Selective activation of Stat3 with adeno-associated viruses allowed to elicit a regenerative response in RGCs but did not improve cell survival [7, 23]. Other signaling pathways contribute to CNTF-induced RGC survival in the optic nerve paradigm such as the Erk1/2 and Akt/PI3K cascades [22, 24, 25]. The duration of cytokine signaling activation is tightly controlled, notably through the negative feedback loop involving *suppressor of cytokine signaling 3* (SOCS3) [8]. Unidentified mechanisms may put a brake on the beneficial effects of CNTF and thereby lead to incomplete axonal regeneration in the damaged visual system. Ultimately, a better knowledge on the mechanisms limiting the regenerative properties of CNTF/Stat3 is needed to enhance vision recovery after RGC injury.

Evidence suggests that *sphingosine 1-phosphate* (S1P) and its receptors may regulate the neuronal growth response associated with retinal inflammation [26]. Indeed, it has previously been shown that the gene expression of the *sphingosine kinase 1* (SphK1), the biosynthesis enzyme of S1P, was dramatically upregulated in isolated RGCs after lens injury-induced inflammation [27]. S1P can bind and activate five G protein-coupled receptors (GPCR), S1PR1-5, which can intracellularly convey negative or positive axonal growth signals. In immature neurons, the activation of S1PR1 activates neurite outgrowth whereas S1PR2 or S1PR5 exerts inhibitory effects *in vitro* [28]. In contrast, the S1P signal transduction mediated by S1PR2 and S1PR5 may result into growth inhibition by activating the RhoA/ROCK pathway [29]. Moreover, our recent results showed that S1PR1 knockdown reduced RGC survival and axon growth after ONC [30]. These effects were correlated with the downregulation of the growth-promoting signaling pathway of mTOR [30] in a subtype of injury-resistant RGCs [31, 32].

**Figure 1:**
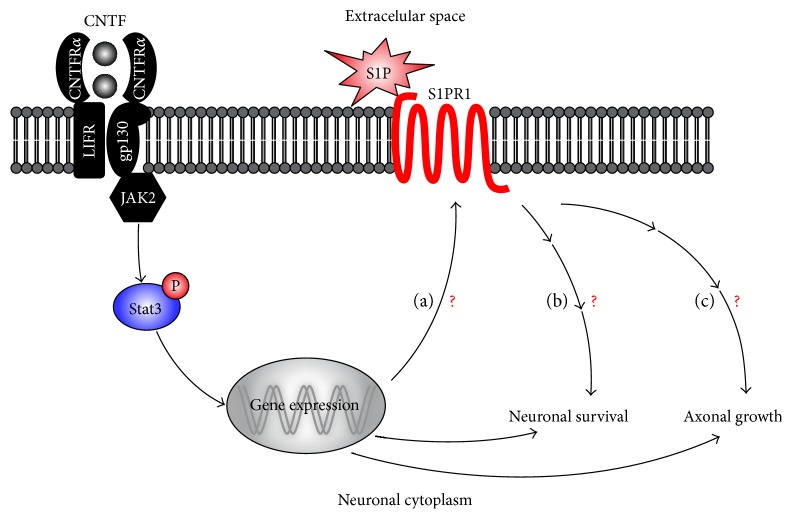
Hypothetical mechanism by which CNTF/Stat3 and S1P/S1PR1 interaction may orchestrate neuronal survival and axonal growth. CNTF binds and activates a heterotrimeric receptor complex, composed of CNTFR*α*, *leukemia inhibitory factor receptor* (LIFR), and gp130, leading to Stat3 phosphorylation (P-Stat3) and activation. (a) P-Stat3-driven transcription may increase the expression of S1PR1 and its translocation to the plasma membrane. The activation of S1PR1 by S1P may trigger downstream growth mechanisms resulting in (b) neuronal survival and (c) axonal growth.

The central role of S1PR1 in Stat3 signaling has been established in tumor and immune cell growth [33] but its importance in CNTF/Stat3-induced growth mechanisms has not been addressed in injured adult neurons (Figure 1). In the current study, we thus undertook to investigate the role of S1PR1 in RGC survival and axonal regeneration after CNTF stimulation and ONC. CNTF cDNA was delivered using an adeno-associated virus (AAV) variant called ShH10 engineered to target Müller glia [16, 34]. On the other hand, S1PR1 was specifically modulated in RGCs with AAV serotype 2 [7, 30, 35]. Our results showed that CNTF or Stat3 upregulated S1PR1 expression in RGCs after ONC. Coinfection experiments with AAV2.shRNA-S1PR1 and ShH10.CNTF provided greater axonal regeneration at long distances past the injury site in the optic nerve. Paradoxically, the rate of surviving RGCs was significantly diminished. The level of active phopho-Stat3 was not affected after S1PR1 silencing in ShH10.CNTF-injected eyes. Together, our data suggest that S1PR1 influences CNTF-induced neuroprotection and axonal regeneration via different mechanisms in the injured visual system.

## 2. Material and Methods

### 2.1. Animals

All surgeries were carried out in 2–4-month-old male C57BL/6 mice (Charles River) in compliance with the guidelines of the Canadian Council on Animal Care guidelines and University Laval Animal Welfare Committee. Animal experiments were conducted in agreement with the ARVO Statement for the Use of Animals in Ophthalmic and Vision Research.

### 2.2. AAV Vector Production and Intravitreal Injections

**Figure 2:**
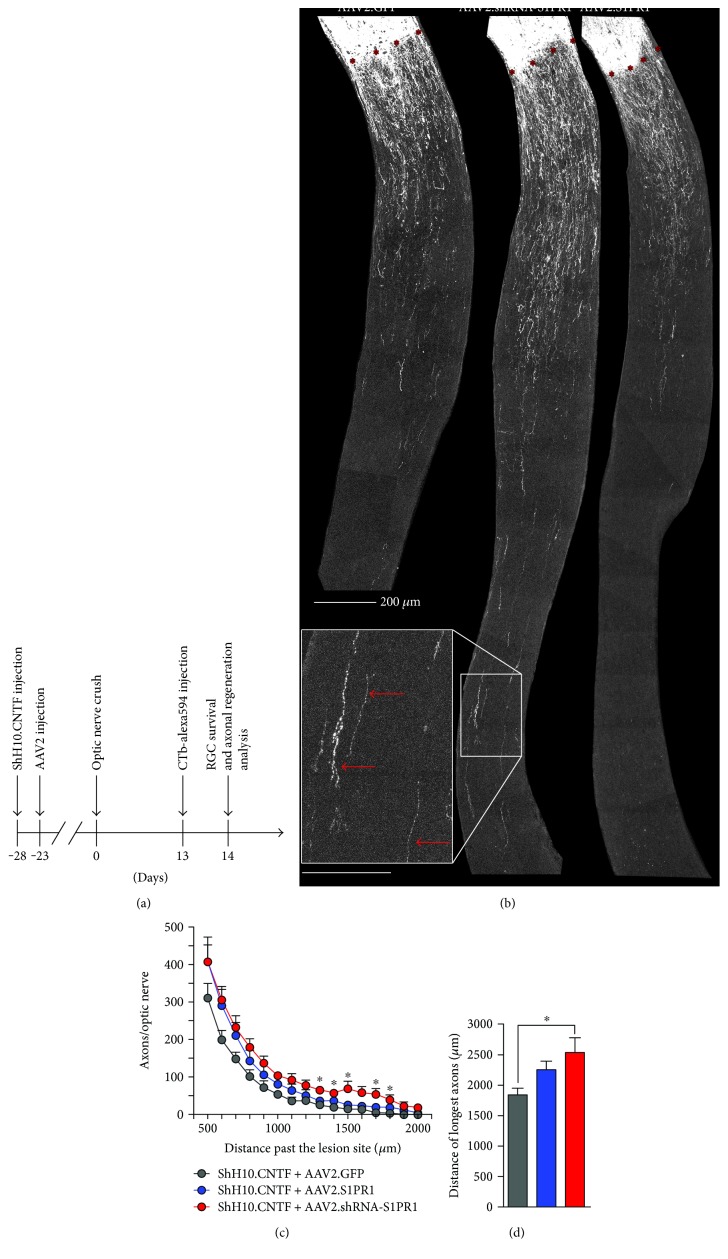
S1PR1 knockdown potentiates CNTF-induced axonal regeneration. (a) Axonal regeneration was visualized on longitudinal sections of optic nerves two weeks after crush injury and 4 weeks after coinfection with ShH10.CNTF and AAV2 vectors. Axons were traced with cholera toxin *β* subunit (CTb) conjugated to Alexa 594 the day before tissue fixation. (b) The infection of retinal cells with ShH10.CNTF and AAV2.shRNA-S1PR1 promoted lengthy axonal regeneration in the optic nerve compared with the ShH10.CNTF/AAV2.GFP combination. (c) Quantitatively, axonal fibers were significantly more numerous between 1300 and 1800 *μ*m past the lesion site with ShH10.CNTF/AAV2.shRNA-S1PR1 (*n* = 6 mice) than with ShH10.CNTF/AAV2.GFP (*n* = 5 mice) treatments (ANOVA, ^∗^*p* < 0.05). ShH10.CNTF/AAV2.S1PR1 did not influence axonal regeneration (*n* = 6 mice). (d) The measurement of the longest axons revealed better growth distances in ShH10.CNTF-/AAV2.shRNA-S1PR1-treated animals than in mice receiving ShH10.CNTF/AAV2.GFP. Scale bars: (b) top = 200 *μ*m; (b) bottom = 100 *μ*m.

The production and efficiency of ShH10.CNTF and AAV2.Stat3-ca have been described before [7, 16]. To modulate S1PR1 expression in RGCs, AAV2 vectors (10^13^ GC/mL) were purchased from Vector Biolabs (Malvern, PA, USA). Gene constructs for S1PR1 silencing and overexpression have previously been validated [30]. Two control vectors were used: AAV2.GFP and ShH10.Empty. ShH10.Empty was produced by omitting cDNA insertion [16]. In order to infect RGCs with AAV2 vectors, 2 *μ*L of each vector was intraocularly injected using a 10 *μ*L Hamilton syringe adapted with a pulled-glass tip like in previous studies [7, 16, 35, 36]. The needle tip was maintained in the vitreous chamber for ~4 min to allow virus diffusion in the eyeball before gentle withdrawal. During intravitreal injections, attention was paid not to damage the lens, the effect of which can result in the release of growth molecules in the eye [9, 37, 38]. For optimal transgene expression, viral vectors were administered 4 weeks before ONC. In some experiments, double injections were realized to infect different retinal cell types with ShH10.CNTF and AAV2.shRNA-S1PR1 or AAV2.S1PR1 or AAV2.GFP, as illustrated in Figure 2(a). For axonal regeneration and RGC survival analysis, 5–7 mice were used per group.

### 2.3. Neuronal Survival Examination

RGC survival and axonal regeneration were assessed in adult mice 2 weeks after ONC. The surgical procedure that was carried out for ONC has been extensively used to study optic nerve axon regeneration in rodents [4, 16, 36, 38–46]. The injury was realized by exposing the optic nerve intraorbitally after longitudinal opening of the optic nerve sheath. A knot was tied around the optic nerve for 20 s with a 9-0 suture at ~0.25–0.5 mm from the rear of the eye. After suture removal, the integrity of the central ophthalmic artery was checked by funduscopic examination. The rate of surviving RGCs was determined after ONC by immunofluorescence on retinal flat-mounts. To this purpose, animals were intracardially perfused with 4% PFA and retinae were flat-mounted. Flattened retinae were then immersed at 4°C in a solution (0.3% Triton-X-100, 5% of bovine serum albumin, and 0.01% sodium azide in PBS) containing a primary antibody (1 : 500; ab18207, Abcam, Toronto, ON, Canada) recognizing *β*3-tubulin, a specific marker for RGCs. *β*3-tubulin-positive RGCs were imaged in the superior, ventral, nasal, and temporal quadrants of the retina using a Zeiss LSM700 confocal microscope equipped with a 20x objective. Image stack acquisition in the ganglion cell layer was led using a step size of 0.5 *μ*m and a resolution of 1024 × 1024 pixels (0.312 *μ*m/pixel). In the 4 retinal quadrants, the number of RGCs was counted in 2 regions of 62,500 *μ*m^2^ each, at 1 and 1.5 mm from the optic disk.

### 2.4. Axonal Regeneration Analysis on Optic Nerve Sections

Axons were anterogradely traced by intraocularly injecting 1.5 *μ*L of 0.5% cholera toxin *β* subunit conjugated to Alexa 594 on day 13 postinjury (CTb, Life Technologies, Burlington, ON, Canada). The following day, the number of growing axons was quantified on longitudinal sections (14 *μ*m) of optic nerves after tissue fixation in 4% paraformaldehyde and cryoprotection in 30% of sucrose. CTb-positive axons were observed in optic nerve sections with a Zeiss Axio Imager Z2 microscope equipped with Zeiss AxioCam MRm camera at 20x magnification. The number of sprouting axons was evaluated at distances ranging from 500 to 2000 *μ*m past the lesion site. Five-six optic nerve sections were analyzed per animal. The number of axons per optic nerve (∑) was calculated using the following formula: ∑_*d*_ = ∏×*R*^2^ × (average number of axons/mm)/*T*. The sum (∑) of axons at a given distance (*d*) was obtained using the average optic nerve radius (*R*) of all optic nerves at 500 *μ*m past the lesion site and a thickness (*T*) of the tissue slices of 14 *μ*m. For statistical analysis with multiple comparisons, ANOVA test was applied followed by Dunnett's post hoc test.

### 2.5. Retinal Section Immunostaining

Mice were euthanized with an overdose of anesthetics and intracardially perfused with PBS and 4% PFA. After cornea and lens removal, eyecups were postfixed in 4% PFA overnight at 4°C, cryoprotected in 30% sucrose, and embedded in OCT compound (Tissue-TEK, Cedarlane, Burlington, ON, Canada). Retinal sections were cut (14 *μ*m) with a cryostat microtome and collected in glass slides. Only central retinal sections that crossed the optic nerve were included in the histological analysis to ensure uniform area sampling in different experimental groups. Immunofluorescent stainings were carried out by incubating retinal sections in a solution (5% of normal goat serum or 5% BSA, 0.3% Triton-X-100 in PBS) of primary antibodies overnight at 4°C. After PBS washes, sections were incubated with the appropriate secondary antibody for 1 h at room temperature. Glass slides were mounted in Mowiol solution (10% Mowiol 4–88 (Millipore, Toronto, ON, Canada) in 100 mM Tris, pH 8.5, 25% glycerol, and 0.1% DABCO). Primary antibodies were rabbit anti-*β*3-tubulin (1 : 1000; ab18207, Abcam), mouse anti-*β*3-tubulin (1 : 1000; G712A, Promega, Madison, WI, USA), rabbit anti-S1PR1 (1 : 50; ASR-011; Alomone Labs, Jerusalem, Israel), rabbit anti-S1PR1 (1 : 200; PA1-1040, Life Technologies), and rabbit anti-phospho-Stat3 (1 : 100; 9131, Cell Signaling, Whitby, ON, Canada). Immunostainings were observed with a Zeiss Axio Imager Z2 microscope equipped with Zeiss AxioCam MRm camera, a Zeiss LSM700 confocal microscope or a Leica SP5 confocal microscope at 40x.

### 2.6. Western Blot Analysis

Immediately after animal sacrifice, retinae were quickly isolated in Eppendorf tubes and snap frozen in liquid nitrogen. Tissues were kept at −80°C until protein lysate preparation in CHAPS lysis buffer containing protease inhibitors (Complete mini, Roche Diagnostics, Laval, QC, Canada). Retinal samples were homogenized and let on ice for 60 min. After centrifugation for 15 min at 15,000 ×g, 4°C, supernatants were retrieved in fresh tubes and used for protein concentration assessment (RC DC Protein Assay; Bio-Rad Laboratories, Mississauga, ON, Canada). Proteins (20 *μ*g/lane) were resolved by electrophoresis on 4–12% polyacrylamide gels and transferred to nitrocellulose membranes. Nitrocellulose membranes were incubated in a blocking solution of 5% bovine serum albumin in 0.2% TBST (0.2% Tween-20 in Tris-base 0.1 M, pH 7.4) for 1 hour at room temperature then incubated with primary antibodies overnight at 4°C. After 3 washings in TBST, the membranes were incubated with horseradish peroxidase-conjugated antimouse or antirabbit secondary antibodies (1 : 10,000–1 : 25,000; Pierce Biotechnology). Primary antibodies were rabbit anti-phospho-Stat3 (1 : 500; 9131, Cell Signaling), rabbit anti-Stat3 (1 : 1000; 9132, Cell Signaling), and mouse anti-GAPDH (1 : 20,000; ab8245, Abcam). Proteins were detected with the WesternSure Premium Chemiluminescent Substrate (Mandel Scientific Company, Guelph, ON, Canada) in a Licor C-Digit blot scanner (Mandel Scientific Company). Band intensities were measured with the ImageJ software (NIH).

### 2.7. Semiquantitative RT-PCR (qRT-PCR)

After cervical dislocation, whole retinae were rapidly dissected, flash frozen in liquid nitrogen, and stored at −80°C until RNA extraction. Retinal RNA was prepared using the RNeasy RNA isolation kit (Qiagen, Toronto, ON, Canada), including a DNase treatment to digest the residual genomic DNA. For reverse transcription, equal amounts of total RNA were transformed to cDNA by using oligo (dT) primers and M-MLV reverse transcriptase (Promega, Madison, WI, USA). 10 ng of cDNA was amplified in the Light Cycler 480 thermocycler (Roche Diagnostics) with the polymerase ready mix (SYBR Green I Master; Roche Diagnostics). The following specific primers were designed to span intronic sequences or cover exon-intron boundaries: *Gapdh* (forward, 5′-CAGCAATGCATCCTGCACC-3′; reverse, 5′-TGGACTGTGGTCATGAGCCC-3′), *S1pr1* (forward, 5′- TCAGGGAACTTTGCGAGTGA-3′; reverse, 5′- AACAGCAGCCTCGCTCAAG-3′), *Sphk1* (forward, 5′-ATACTCACCGAACGGAAGAAC-3′; reverse, 5′- ATTAGCCCATTCACCACCTC-3′), and *Sphk2* (forward, 5′-GCTTTACGAGGTGCTGAATG-3′; reverse, 5′-AGAAGCGAGCAGTTGAG-3′). For relative quantification of gene expression, mRNA levels were normalized to GAPDH using the comparative threshold cycle (∆∆^CT^) method and a control sample was used to calculate the relative values. Each reaction was done in triplicate using 3 mice per condition.

### 2.8. Statistical Analysis

Graph values represent mean ± standard error of the mean (SEM). Statistical analyses were conducted with the GraphPad Software, Prism 5, by applying one-way analysis of variance (ANOVA) tests followed by Dunnett's post hoc test in experiments comprising more than 2 groups of mice. Quantitative analyses were done in blind.

## 3. Results

### 3.1. AAV-Mediated CNTF/Stat3 Activation in RGCs Increases S1PR1 Expression after Injury

**Figure 3:**
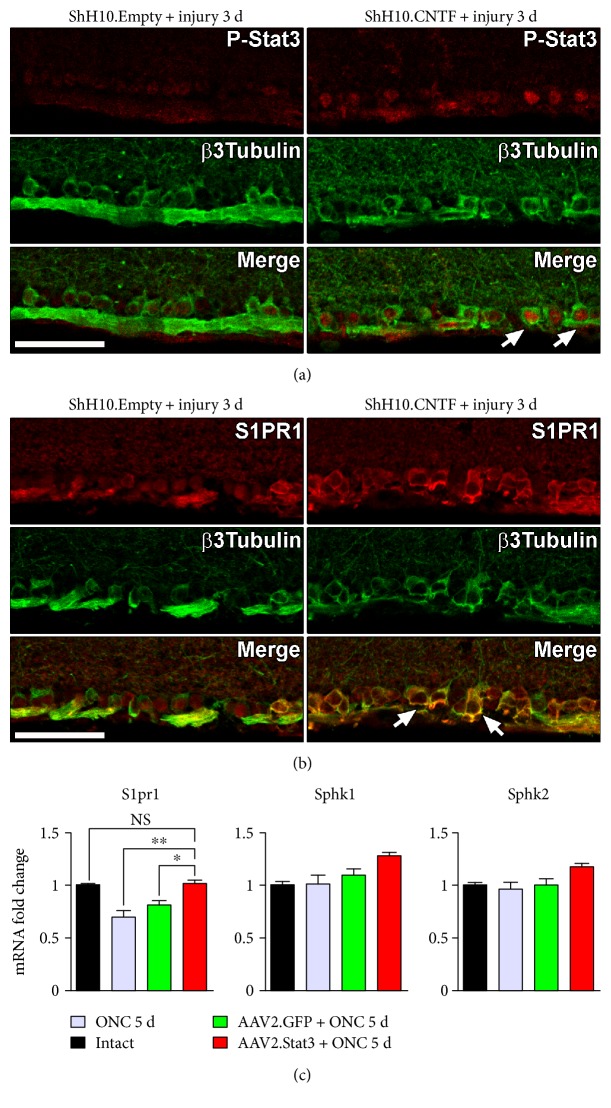
Modulation of S1PR1 expression by the CNTF/Stat3 pathway in optic nerve-injured retinae. (a, b) S1PR1 expression changes were monitored 3 d after ONC in retinae infected with ShH10.CNTF or ShH10.Empty, a control vector that was deprived of cDNA sequence. ShH10 viruses preferentially infected Müller glia in the retina [16]. P-Stat3 and S1PR1 were markedly increased by ShH10.CNTF in RGC somata identified using *β*3-tubulin as a specific marker. (c) Five days after ONC, qRT-PCR measurements showed that the infection of RGCs with AAV2.Stat3 significantly increased the mRNA level of *S1pr1* compared with control AAV2.GFP vector. ShH10 and AAV2 viruses were intravitreally injected 4 weeks before ONC. Three mice were analyzed/grouped. Statistics: one-way ANOVA; ^∗^*p* < 0.05, ^∗∗^*p* < 0.01; NS: not significant. Scale bar: 50 *μ*m.

We have previously reported that ONC caused S1PR1 downregulation in RGCs [30]. The expression of S1PR1 is required to keep RGCs alive and to allow spontaneous axonal sprouting in the optic nerve after crush lesion. Here, we sought to determine if the expression of S1PR1 was influenced by CNTF/Stat3 activation in injured RGCs. To address this question experimentally, we monitored S1PR1 expression changes after AAV-mediated CNTF/Stat3 signaling activation in RGCs. The injection of ShH10.CNTF, a virus preferentially infecting Müller glia, allowed to detect a brighter signal for P-Stat3 and S1PR1 in injured RGCs compared with control ShH10.Empty virus (Figures 3(a) and 3(b)). Stat3 is a key signaling component involved in CNTF-induced axonal growth in the injured optic nerve [7, 16, 21–23]. However, as other signaling cascades are activated by CNTF (e.g., Erk1/2), the mechanism controlling S1PR1 upregulation was uncertain [16]. Therefore, mice were infected with AAV2.Stat3 in order to selectively assess the influence of Stat3 on S1PR1 expression in injured RGCs. In our previous experiments, we found that selective infection of RGCs with AAV2.Stat3 construct was sufficient to promote axonal growth in the crushed optic nerve [7]. Five days after ONC injury, qRT-PCR measurements showed that AAV2.Stat3 significantly increased the mRNA level of *S1pr1* in whole retinal lysates compared with AAV2.GFP (Figure 3(c)). In AAV2.Stat3-treated retinae, the mRNA level of *S1pr1* was not different from that measured in intact retinae. Interestingly, the level of *sphingosine kinase 1* (*Sphk1*) and *Sphk2* mRNA, the rate-limiting enzymes for S1P synthesis, was not significantly affected by ONC and AAV2-mediated Stat3 overexpression in RGCs (Figure 3(c)). These observations suggest that retinal production of S1P is not limiting for the activation of S1PR1 after injury. Together, these results suggest that CNTF/Stat3 may prevent injury-induced S1PR1 downregulation in RGCs. The function of S1PR1 was then analyzed in RGC survival and axonal regrowth after CNTF stimulation.

### 3.2. S1PR1 Knockdown Improves Axonal Regeneration after CNTF Stimulation

We have previously reported that S1PR1 knockdown reduced spontaneous axonal outgrowth after ONC, presumably as a result of increased RGC death [30]. Here, we set out to evaluate the effects of S1PR1 in CNTF-induced axonal regeneration. In this aim, adult mice were intravitreally injected with ShH10.CNTF first and 5 d later with AAV2.GFP, AAV2.S1PR1, or AAV2.shRNA-S1PR1 (Figure 2(a)). Axons were traced by injecting cholera toxin *β* subunit coupled to Alexa 594 (CTb-594) in the vitreous space of axotomized eyes the day preceding anatomical analysis of growth. Combined with CNTF stimulation, AAV2.shRNA-S1PR1 increased the distance of axonal regeneration stimulated by CNTF in crushed optic nerves compared with AAV2.GFP or AAV2.S1PR1 treatments (Figure 2(b)). Quantitatively, the number of regenerating axons was significantly higher in AAV2.shRNA-S1PR1-injected mice than in other groups at distances ranging from 1300 *μ*m to 1800 *μ*m past the lesion site (Figure 2(c)). The measurement of the longest axons for each optic nerve section also revealed an increase in growth distance when AAV2.shRNA-S1PR1 was administrated with respect to other AAV2 treatments (Figure 2(d)). Together, these results suggest that blocking S1PR1 expression improves CNTF-induced axonal growth in the optic nerve.

### 3.3. CNTF-Induced RGC Survival Depends on S1PR1 Expression

**Figure 4:**
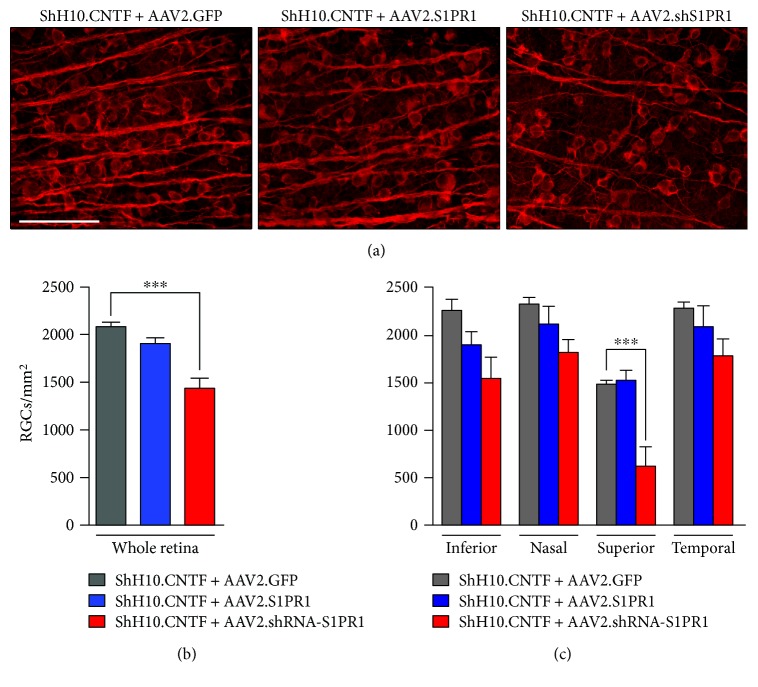
S1PR1 knockdown alters CNTF-induced RGC survival after ONC. (a) Two weeks after ONC, surviving RGCs were observed in retinal flat-mounts after immunofluorescent staining for *β*3-tubulin. Less RGCs were visible in retinae infected with AAV2.shRNA-S1PR1 and ShH10.CNTF (*n* = 5 mice) than in mice injected with ShH10.CNTF/AAV2.GFP (*n* = 7 mice) or ShH10.CNTF/AAV2.shRNA-S1PR1 (*n* = 5 mice). (b) Quantitatively, the average number of surviving RGCs was statistically lower in whole retinae transduced with ShH10.CNTF/AAV2.shRNA-S1PR1 than in the two other groups of animals (ANOVA, ^∗∗∗^*p* < 0.001). (c) The reduction of RGC survival caused by ShH10.CNTF/AAV2.shRNA-S1PR1 was the most pronounced in the superior quadrant of the retina (ANOVA, ^∗∗∗^*p* < 0.001). Scale bar: 100 *μ*m.

RGC survival was examined by staining retinal flat-mounts for *β*3-tubulin 2 weeks after ONC. We have previously shown that RGC transduction with S1PR1 shRNA caused neuronal loss [30], although in a separate study, we found that intraocular ShH10.CNTF delivery dramatically enhanced RGC survival [16]. Here, the combination of the 2 viral vectors was tested on RGC survival. Strikingly, despite CNTF secretion from Müller glia, AAV2-mediated S1PR1 downregulation caused a 31% decrease in the density of surviving RGCs relative to AAV2.GFP control treatment (Figures 4(a) and 4(b)). The most pronounced loss of RGCs occurred in the superior quadrant (Figure 4(c)), where the number of RGCs was decreased by 58% compared to control AAV2.GFP-/ShH10.CNTF-treated retinae. S1PR1 overexpression with AAV2.S1PR1 had, however, no influence on the number of *β*3-tubulin-labelled RGCs. The lower rate of surviving RGCs obtained with AAV2.shRNA-S1PR1 is paradoxical in regard to the increased axonal regeneration observed after ShH10.CNTF administration (Figure 2(c)). This apparent discrepancy may be explained by the fact that the survival of regenerating RGCs may not require S1PR1 signaling activation (see Discussion).

### 3.4. S1PR1 Does Not Influence CNTF-Induced Stat3 Activation

**Figure 5:**
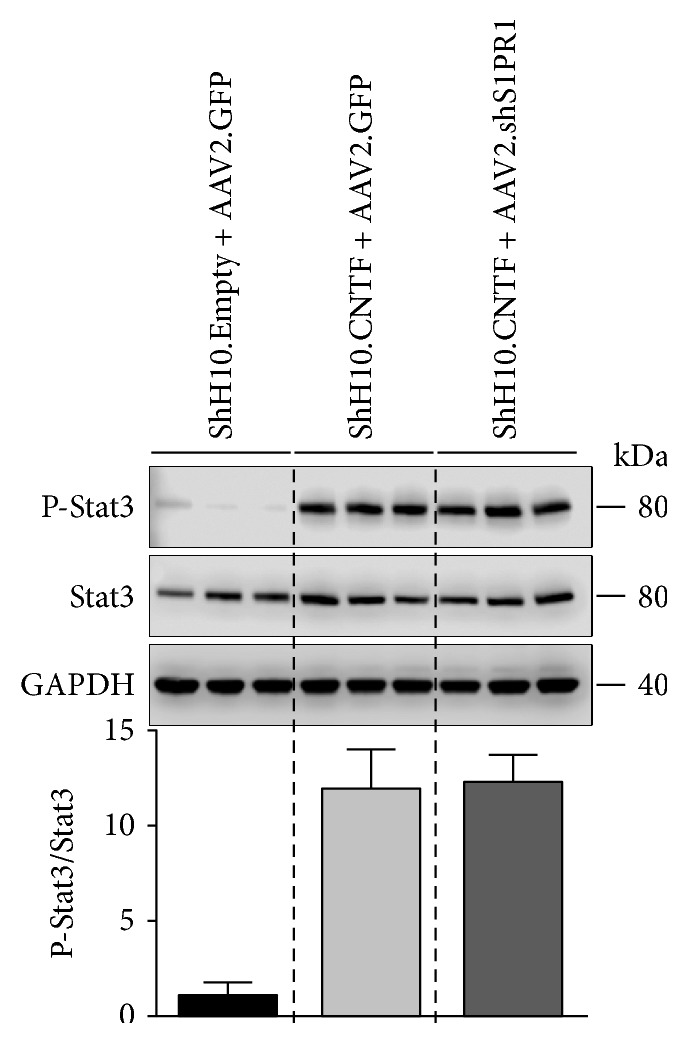
The expression of P-Stat3 is not changed by S1PR1 silencing after CNTF stimulation. The expression of P-Stat3 was assessed by western blotting in protein lysates (20 *μ*g) from retinae treated with ShH10 and AAV2 viruses. P-Stat3 and Stat3 blots were quantified by densitometry using the ImageJ software (NIH). The level of P-Stat3/Stat3 was not significantly different between mice treated with ShH10.CNTF/AAV2.shRNA-S1PR1 and ShH10.CNTF/AAV2.GFP. Three mice were analyzed for each group.

We investigated the effect of S1PR1 silencing on the level of phosphorylated, active Stat3 (P-Stat3) in ShH10.CNTF-treated retinae. P-Stat3 expression was not affected by AAV2.shRNA-S1PR1 as shown by western blot analysis (Figure 5). This suggests that the CNTF/Stat3 axis is independent of S1PR1 expression in RGCs.

## 4. Discussion

Our data show that S1PR1 expression can be controlled by the Stat3 transcription factor in injured RGCs. CNTF-induced axonal regeneration was potentiated in the optic nerve by retinal infection with AAV2.shRNA-S1PR1. Unexpectedly, RGC cell survival was reduced after silencing S1PR1 and the overexpression of CNTF in the retina. To us, this marked increase in cell death points at an important role for S1PR1 in the mechanisms underlying CNTF-induced survival. To explain the negative and positive effects of AAV2.shRNA-S1PR1 on cell survival and axonal regeneration, respectively, we propose that S1PR1 may play deleterious and beneficial effects on distinct RGC subpopulations.

### 4.1. S1PR1 Is Involved in CNS Axonal Regeneration

Silencing S1PR1 enhanced the axonal growth induced by CNTF in the optic nerve. This speaks in favor of an inhibitory function for S1PR1 in axon regeneration in the injured CNS. However, the fact that axonal growth was only significantly increased at relatively long distances from the injury site suggests that S1PR1 silencing may promote axonal branching rather than individual axon growth. Our previous observations revealed that axonal branching was rare after CNTF stimulation but more frequent when Stat3 was selectively increased in RGCs [7]. CNTF can activate other signaling cascades such as that of Erk1/2 and Akt that may limit axonal branching [7]. The possible induction of axonal branching by the combination of AAV2.shRNA-S1PR1 and ShH10.CNTF treatments may thus result from the regulation of Stat3-independent mechanisms. This hypothesis is consistent with the lack of change in P-Stat3 activation after AAV2.shRNA-S1PR1 infection. In contrast to the present data, we have recently reported that S1PR1 silencing reduced spontaneous axonal sprouting after ONC [30]. The opposite effects of S1PR1 shRNA on spontaneous and CNTF-induced axonal growth could depend on the modulation of distinct molecular mechanisms. After ONC, spontaneous axonal sprouting is extremely modest and may require endogenous mTOR signaling activation. The mTOR pathway is recognized as a major mechanism by the manipulation of which massive axonal growth can be generated in the optic nerve [6]. Interestingly, our previous study revealed that S1PR1 knockdown was associated with mTOR signaling downregulation in injured RGCs [30]. As mTOR and CNTF/Stat3 orchestrate independent growth mechanisms in neurons [8], it may be that S1PR1 exerts positive effects in mTOR-driven axonal sprouting and negative effects in CNTF/Stat3-induced axonal regeneration.

In a different context, Toman et al. have previously shown that S1PR1 had positive effects on neurite outgrowth in cell cultures [28]. The upregulation of S1PR1 expression conferred on S1P the ability to stimulate PC12 process extension [28] by simultaneously activating Rac-GTPase and by inhibiting RhoA [28]. These findings are in contradiction with those presented in the current study. However, obvious experimental differences may very well explain why our results showed an opposite involvement of S1PR1 in axonal growth. Contrary to Toman et al. who used a neuronal cell line (PC12) and embryonic neurons in culture, we studied the growth of adult RGCs that constitute a mature neuron population. In addition, Toman et al. induced neurite outgrowth with NGF, a neurotrophic factor that is not effective in activating RGC axonal regeneration. Consequently, the experimental conditions selected to investigate the role of S1PR1 in neurite outgrowth may strongly influence the outcome. However, in agreement with our current results, the blockade of S1PR1 with the FTY720 compound improved neurite outgrowth and facial nerve regeneration after axotomy [47]. As FTY720 does not exclusively block S1PR1, other receptors may be involved in its regenerative properties [48]. In addition, nonneuronal cells (e.g., immune cells) may mediate the effects of this pharmacological blocker indirectly. By using AAV2 vectors whose tropism is selective to RGCs in the eye, we did not modify S1PR1 expression in other cell types including immune cells. The molecular mechanisms underlying axonal growth potentiation with AAV2.shRNA-S1PR1 were not elucidated in our study. In the ONC paradigm, the RGC growth response to CNTF stimulation is highly dependent on Stat3 activation [7, 21–23]. This is why we examined this particular pathway by western blotting. By itself, the elevation of P-Stat3 in RGCs is sufficient to trigger axonal extension in the distal part of the injured optic nerve [7, 22, 23]. However, we found that AAV2-mediated S1PR1 downregulation did not significantly enhance CNTF-induced Stat3 activation in retinal lysates, making crosstalk between S1PR1 and CNTF/Stat3 signalings unlikely in the experimental setup that we have used.

### 4.2. S1PR1 Is a Major Regulator of RGC Neuroprotection

AAV2-mediated S1PR1 downregulation impaired CNTF-induced survival after injury. To a similar extent, without CNTF stimulation, we have previously observed that S1PR1 shRNA exacerbated ONC-induced RGC death [30]. In addition, the reduction of S1PR1 expression was associated with the downregulation of mTOR activation in *α*RGCs. This cell type belongs to a small group (15–20% of total RGCs) of injury-resistant RGCs that possess a remarkable resistance to injury-induced cell death [31]. The predominant role of mTOR in the extraordinary survival ability of *α*RGCs was demonstrated with the administration of the pharmacological inhibitor rapamycin. The injection of this compound dramatically reduced the rate of surviving RGCs after injury [6]. The downregulation of mTOR resulting from the action of S1PR1 shRNA may thus be responsible for the loss of injury-resistant RGCs, such as *α*RGCs. Indeed, RGC stimulation with CNTF may be inefficient at rescuing injured cells when the mTOR pathway is blocked since Stat3 and mTOR are thought to operate independently in RGC neuroprotection [49]. In addition, the loss of RGCs was not correlated with a lower level of P-Stat3 activation in retinae infected with AAV2.shRNA-S1PR1, suggesting that the CNTF/Stat3 axis is relatively inconsequential for RGC survival. In agreement with this, it has previously been reported that selective Stat3 activation in RGCs did not increase the level of neuronal survival after ONC [7, 23]. In contrast, Erk1/2 activation plays a central role in RGC survival [22, 25]. Different growth factors, including CNTF [16, 24] and *brain-derived neurotrophic factor* (BDNF) [50] or *vascular endothelial growth factor* (VEGF) [51], promote RGC survival via Erk1/2 phosphorylation. Crosstalk between S1P-S1PR1 and growth factor signaling has been evoked in nonneuronal cells like vascular endothelial cells. For instance, S1P inhibits VEGF signal transduction by causing VEGFR2 internalization in vascular endothelial cells [52]. Therefore, one cannot exclude the possibility that growth factor receptors involved in RGC survival, such as VEGFR2, are regulated by S1PR1.

### 4.3. Conclusion

The concomitant decrease in the density of surviving RGCs and the enhanced axonal growth in the optic nerve following S1PR1 knockdown suggests that (1) S1PR1 expression is a major regulator of RGC survival induced by CNTF and (2) S1PR1 restricts axonal growth in a subset of RGCs that are sensitive to CNTF/Stat3 growth stimulation. Further investigations will be necessary to discriminate the subset of RGCs whose survival depends on S1PR1. Ultimately, functional RGC repair may be obtained with treatments that enable the survival and regeneration of a wide variety of RGC subtypes, encoding different aspects of vision.
